# Current State of “Omics” Biomarkers in Pancreatic Cancer

**DOI:** 10.3390/jpm11020127

**Published:** 2021-02-14

**Authors:** Beste Turanli, Esra Yildirim, Gizem Gulfidan, Kazim Yalcin Arga, Raghu Sinha

**Affiliations:** 1 Department of Bioengineering, Marmara University, 34722 Istanbul, Turkey; turanlibeste@gmail.com (B.T.); essyildirim@gmail.com (E.Y.); gizemgulfidn@gmail.com (G.G.); 2 Turkish Institute of Public Health and Chronic Diseases, 34718 Istanbul, Turkey; 3 Department of Biochemistry and Molecular Biology, Penn State College of Medicine, Hershey, PA 17033, USA

**Keywords:** pancreatic cancer, systems biology, omics, biomarker, genomics, transcriptomics, proteomics, metabolomics, glycomics, metagenomics, personalized medicine

## Abstract

Pancreatic cancer is one of the most fatal malignancies and the seventh leading cause of cancer-related deaths related to late diagnosis, poor survival rates, and high incidence of metastasis. Unfortunately, pancreatic cancer is predicted to become the third leading cause of cancer deaths in the future. Therefore, diagnosis at the early stages of pancreatic cancer for initial diagnosis or postoperative recurrence is a great challenge, as well as predicting prognosis precisely in the context of biomarker discovery. From the personalized medicine perspective, the lack of molecular biomarkers for patient selection confines tailored therapy options, including selecting drugs and their doses or even diet. Currently, there is no standardized pancreatic cancer screening strategy using molecular biomarkers, but CA19-9 is the most well known marker for the detection of pancreatic cancer. In contrast, recent innovations in high-throughput techniques have enabled the discovery of specific biomarkers of cancers using genomics, transcriptomics, proteomics, metabolomics, glycomics, and metagenomics. Panels combining CA19-9 with other novel biomarkers from different “omics” levels might represent an ideal strategy for the early detection of pancreatic cancer. The systems biology approach may shed a light on biomarker identification of pancreatic cancer by integrating multi-omics approaches. In this review, we provide background information on the current state of pancreatic cancer biomarkers from multi-omics stages. Furthermore, we conclude this review on how multi-omics data may reveal new biomarkers to be used for personalized medicine in the future.

## 1. Introduction

Pancreatic cancer is one of the most fatal malignancies and the seventh leading cause of cancer-related deaths considering both sexes worldwide according to the latest global cancer statistics reported in 2018 [1]. Pancreatic cancer has a difficult diagnosis at an early stage and a 5 year survival rate of 10% at the time of diagnosis in the United States, where the poor survival rates have hardly changed for almost 40 years since most patients reporting to the hospital have either unresectable or metastatic disease. Only 10.8% of these patients are at a locally advanced stage at the time of diagnosis [2,3]. Unfortunately, pancreatic cancer is projected to become the third leading cause of cancer deaths in the future [1].

It is a great challenge to intervene at the early stages of pancreatic cancer that is in initial diagnosis or postoperative recurrence because of the difficulties in early diagnosis and inadequacy in precise prognostic biomarkers, and this challenge may result in undesirable overdiagnosis and/or overtreatment, causing the high mortality rate [4,5,6,7].

Pancreatic cancer can be divided into two large groups; (a) endocrine pancreatic tumors, including gastrinoma, glucagonoma, and insulinoma, and (b) exocrine (non-endocrine) pancreatic tumors, including adenoma, ductal adenocarcinoma, acinar cell carcinoma, cystadenocarcinoma, adenosquamous carcinoma, signet ring cell carcinoma, hepatoid carcinoma, colloid carcinoma, undifferentiated carcinoma, pancreatoblastoma, and pancreatic mucinous cystic neoplasm [8,9]. Most of the pancreatic cancers are exocrine types—namely, ductal adenocarcinoma, which comprises 80–90% of all pancreatic cancers; whereas endocrine (neuroendocrine) pancreatic tumors are rare with 1–2% of all pancreatic cancers [7].

Moreover, pancreatic neoplasms can be categorized by their gross appearance as solid, cystic, or intraductal. The solid pancreatic tumors contain pancreatic ductal adenocarcinoma (PDAC), neuroendocrine (islet cell) neoplasms, acinar cell carcinomas, and pancreatoblastoma. The cystic types of pancreatic tumors tend to be less aggressive and include mucinous cystic neoplasms, serous cystadenoma, intraductal papillary mucinous neoplasms, and solid-pseudopapillary neoplasms [10]. Pancreatoblastoma is mostly observed in childhood, and it has a poor prognosis if an adult is diagnosed with it. Mucinous cystic neoplasms consist of a range from benign to malignant [7].

The World Health Organization (WHO) classifies the morphological variants of PDAC differently from the conventional pancreatic adenocarcinoma classification. These variants have different histological features besides molecular signatures and prognosis. According to WHO, the different subtypes of PDAC are adenosquamous carcinoma, colloid/mucinous carcinoma, undifferentiated/anaplastic carcinoma, signet ring cell carcinoma, medullary carcinoma, and hepatoid carcinoma [11].

Like most cancer types, pancreatic cancer has also several known risk factors, such as cigarette smoking, diabetes, obesity, lack of physical activity, and chronic pancreatitis [12,13]. Currently, computed tomography (CT), magnetic resonance imaging (MRI), endoscopic ultrasound (EUS), positron emission tomography (PET), and other imaging methods are used in the diagnosis and prognosis of pancreatic cancer [12,13,14].

Unsurprisingly, early detection of PDAC by effective screening approaches is crucial to improve a better prognosis of the disease. The absence of clinical symptoms in the early stage of pancreatic cancer could lead to a delay in confirmed diagnosis even though tumor biomarkers and imaging techniques are being developed. Therefore, using circulating biomarkers for primary screening and its combination with imaging and histopathologic results might be the future strategy for diagnosing PDAC. Candidate circulating biomarkers in PDAC are not limited to circulating tumor cells (CTC) but also consist of metabolites, cell-free DNA and non-coding RNA, exosomes, autoantibodies, and inflammatory or growth factors, which are recently summarized [15]. The presence of CTCs in the blood usually correlates with the systemic spread of the tumor, and the characteristics of these CTCs could be used as potential biomarkers. Moreover, the challenging tasks of CTC isolation and detection are being overcome [16,17], and the emerging area of profiling CTCs has been recognized in prognosis of pancreatic cancer [18].

**Figure 1:**
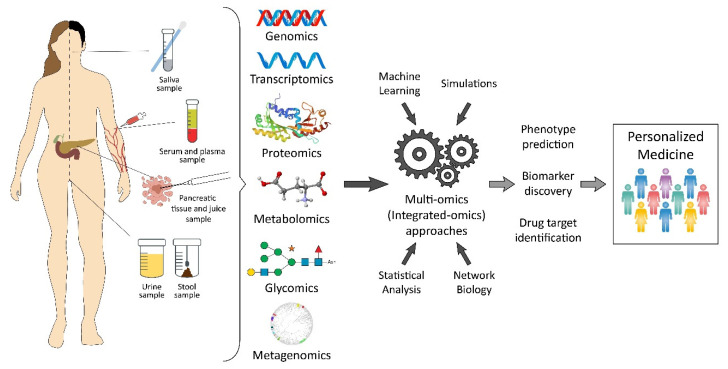
A conceptual review of pancreatic cancer biomarkers from a variety of “omics” levels.

Sample source is very critical in the identification of biomarkers for the detection and diagnosis of early-stage pancreatic cancer [19]. The pancreas is located in the back of the abdomen and is surrounded by the stomach, small intestine, liver, and spleen, so it becomes a big challenge in getting a biopsy. The most common way to get pancreatic tumor samples is by fine-needle aspiration (FNA). However, a core needle biopsy using a larger needle than an FNA can provide a larger sample, often useful for molecular profiling. These biopsies can be taken with an EUS. Other biopsy types, like brush biopsy or forceps biopsy, can be done during an endoscopic cholangiopancreatography (ERCP). However, body fluids such as blood, cyst fluid, pancreatic juice, bile, as well as urine are characteristically enriched with biomarkers that can be a potential source of diagnostic, predictive, and/or prognostic biomarkers in PDAC. As a source of pancreatic cancer biomarker, saliva has also been used. In omics biomarker studies, blood is a frequently preferred sample source due to its easy accessibility, noninvasiveness, and cost-effectiveness [20]. As an alternative rich source for the discovery of biomarkers, pancreatic juice has recently been identified. Pancreatic juice contains pancreatic cancer-specific markers such as DNA, RNA, proteins, and cancer cells, but the collection procedure for this sample source is invasive [19]. Although urine contains limited protein, DNA, and RNA, it can be considered as an ideal source sample for proteomic and genomic biomarkers [21]. Furthermore, accurate staging is very important for providing appropriate treatment. The majority of the time, surgical excision is used for treatment, and traditional chemoradiotherapy has very restricted effectiveness, despite the development of novel therapy options [7]. In this review, we present a systems-level outlook of PDAC biomarkers from different “omics” levels (Figure 1) as well as a comprehensive overview of methodology and sampling used in biomarker studies for PDAC (Table 1).

## 2. Recent Insights from Different Omics Levels

Despite the substantial advancement in pancreatic cancer research, there has not been any remarkable reduction in the mortality-to-incidence ratio. This is mainly a result of the limited early diagnostic characteristic symptoms and reliable biomarkers, besides the unresponsiveness to the treatments due to the tumor heterogeneity, plasticity, and the aggressive metastasis that presents in more than 50% of the diagnosed patients [22].

Systems biology studies of pancreatic cancer rely on the integration of omics data from different biological levels. With the frequently arising challenges regarding cancer diagnosis and treatment—mainly due to its complex pathogenic landscape and cellular heterogeneity—the holistic view provided by the systems biology approach allowed for having a global understanding of the mechanisms of the disease and gaining more insight toward diagnostic or prognostic biomarkers and drug target discovery [23,24].

Likewise, systems biology also augments current diagnosis and therapy options. Aggressiveness and chemoresistance of PDAC are caused by the desmoplastic reactions induced by immune cells, stromal cells, neural cells, and the extracellular matrix surrounding and forming the bulk of the tumor mass. Therefore, single-cell sequencing may shed a better insight into cellular differences. Moreover, altered metabolism is caused by limited delivery of the needed oxygen and nutrients in such a hypoxic and acidic microenvironment; a direct impact on the drug delivery mechanisms is common [25,26].

## 3. Genomic Signatures

Next-generation sequencing (NGS) provides support for the early diagnosis and screening of PDAC as well as many other diseases. Genomics techniques may assist in the early diagnosis of pancreatic cancer in patients with specific alleles that predispose them to cancer development. Different potential biomarkers discovered by genomics methods can be categorized as chromosomal aberrations, driver changes, single nucleotide polymorphisms (SNPs), or copy-number alterations.

Previous studies pointed out the most prominent genetic features of PDAC, such as oncogenic activation of K-RAS, which is a standard feature in more than 90% of the patients, and with the early onset mutation of that gene, it is considered a critical driver of PDAC initiation and progression [27]. Along with the oncogenic activation, inactivating mutations of the tumor suppressor gene CDKN2A/2B are also observed in more than 80% of the early-stage lesions, while later stages of PDAC exhibit inactivating mutations and deletions of tumor suppressor genes most prominently including TP53 and SMAD4 [28].

Metabolic reprogramming is considered a prominent hallmark of PDAC. Therefore, tackling this aggressive cancer might be possible through establishing a clear understanding regarding its metabolism in addition to genomics [29]. Recent studies have shown the crucial role of both glucose and glutamine metabolism in the progression of PDAC tumors that are regulated by the K-RAS oncogene to maintain tumor growth [30,31,32]. Inducible oncogenic K-RAS mouse model of PDAC showed—in addition to being a key driver of PDAC initiation—that it plays a central role in rewiring the tumor glucose metabolism by stimulating the glucose uptake and driving glycolysis intermediates toward nonoxidative pentose phosphate pathways [31]. It was also reported that the PDAC cells maintain the tumor growth by relying on the distinct pathway of glutamine metabolism and that this reprogramming is mediated by K-RAS [30].

Therefore, not only genomics biomarkers but also network reconstructions [33], including different omics levels, become an essential tool for exploring the disease under the systems biology perspective. Network models and computational platforms for integrating and analyzing these data, as well as investigating more thoroughly into these networks by simulations, are prominent efforts.

## 4. Coding and Noncoding RNA Signatures of Pancreatic Cancer

Initial transcriptome studies were performed for analysis of the mRNA profiles, which focused on protein-coding genes in PDAC. Thereafter, researchers compared gene expression levels between tumors and normal pancreas tissues and determined the genes with altered expression profiles in the disease state; this assisted in discovering potential diagnostic or prognostic biomarkers [34]. Over the years, microarray and RNAseq technology have been utilized not only to obtain coding but also non-coding RNA signatures. Although transcriptomic studies of non-coding RNAs are mainly focused on microRNAs (miRNA) and long non-coding RNAs (lncRNAs), other non-coding RNA types such as piwi interacting RNA (piRNAs), circular (circRNAs), small nucleolar RNA (snoRNA), and small nuclear RNA (snRNA) [35] are also promising biomarker candidates as they are quantitatively assessed, providing opportunities for noninvasive and early diagnosis of PDAC [20].

miRNAs involve in the expression of posttranscriptional regulatory mechanisms [36] and act as oncogenes or inhibit tumor suppressors in PDAC. Overexpression of the oncogene miRNAs (oncomir) increases in tumor progression, while tumor suppressors inhibit cell proliferation and induce apoptosis [37] by inactivating TP53, P16, and SMAD4 in PDAC [38]. miRNAs have the advantage of being stable in serum, hence these show remarkable potential as diagnostic biomarkers or a prognostic tool for noninvasive detection and convenient screening [39]. Therefore, the use of miRNA expression profiling has gained importance for the early detection of cancer [40,41].

Dysregulation of miRNAs in PDAC has been investigated not only in pancreatic tumors but also in blood samples, pancreatic juice, stool, urine, and saliva [39,42]. In several studies, the expression levels of miR-21, miR-155, and miR-196 have been reported to be upregulated in PDAC [43,44,45,46]. The higher concentration of miR-155 and miR-210 in the sera of pancreatic cancer patients as compared to normal healthy individuals has been proposed as a potential diagnostic marker in the early stages of pancreatic cancer [47,48]. Moreover, miR-155 and miR-21 were also found to have increased expression in pancreatic juices, while expressions are linked with histological progression characteristics [49]. In addition, the evaluation of more than 700 miRNAs in a study using blood samples compared between pancreatic cancer patients and healthy individuals emphasized miR-1290 as a promising biomarker [50]. Likewise, multiple studies have proposed not only miR-21, miR-155, miR-196, and miR-1290 but also miR-200, miR-18a, miR-210, miR-192, miR-22, miR-642b, miR-885-5p, and miR-375 as candidate biomarkers for PDAC patients [47,51,52,53,54,55]. Another comparison between cancer patients and healthy individuals clearly showed a distinct miRNA expression profile that included upregulation of miR-21, miR-23a, miR-31, miR-100, miR-143, miR-155, miR-2214, and downregulation of miR-148a, miR-375, and miR-217 [43].

The combination of various biomarkers such as CA19-9 with miR-16 and miR-196a provoked distinct improvement to distinguish between PDAC patients and healthy controls [56]. Similarly, the miR-27a-3p expression profile coupled with CA19-9 differentiated PDAC patients and healthy controls with a sensitivity and specificity of more than 80% [57,58]. Among diagnostic features of miRNAs, poor survival in PDAC patients was determined regarding overexpression of miR-221/222 and miR-744 levels in tumor tissue and plasma, respectively, as well as low-expression levels of miR-218 and miR-494 in tumor tissue [59,60,61,62].

In addition to microRNAs, other non-coding RNAs—such as long non-coding RNAs (lncRNAs), small nuclear RNAs (snRNAs), or circular RNAs (circRNAs)—have also been identified that might have potential as diagnostic or prognostic markers for PDAC. Long non-coding RNAs (lncRNAs) consist of more than 200 nucleotides, and some of them are circulating in body fluids which makes them promising markers for disease detection [63]. Although the biological functions of lncRNAs are not fully understood, the expression of lncRNAs (HOTAIR, MALAT-1, GAS5, MEG3, HULC, BC008363, and HSATII) showed significant alterations in pancreatic cancer cell lines. Besides, HOTAIR and PVT1 had higher concentrations in saliva in PDAC patients than saliva taken from healthy individuals. Therefore, these lncRNAs in saliva offer a potential noninvasive detection method for PDAC [35]. To date, U2snRNA, which is overexpressed in PDAC, has been the only reported snRNA biomarker in PDAC patients [64].

Circular RNAs (circRNAs), as another type of non-coding RNAs, have drawn increased attention through their regulatory roles in cancer. Generally, these are generated from precursor mRNA (pre-mRNA) by canonical splicing and head-to-tail back splicing, which makes them circular. Moreover, their structure without a polyA tail makes circRNAs favorably insensitive to ribonuclease and more desirable as clinically useful biomarkers. These function as miRNA sponges and overwhelm the ability of the miRNA to bind its mRNA targets [65]. Therefore, the associations of miRNAs and circRNAs with their potential regulatory role were also investigated in PDAC. For instance, hsa_circ_0005785 is potentially able to bind miR181a and miR181b as “oncomiRs” in pancreatic cancer, while miR-181a plays a critical role in regulating cancer growth and migration [66]. In another study, two upregulated circRNAs (hsa_circ_0001946, hsa_circ_0005397) and five downregulated circRNAs (hsa_circ_0006913, hsa_circ_0000257, hsa_circ_0005785, hsa_circ_0041150, and hsa_circ_0008719) were proposed as biomarkers after microarray analysis. They also validated the expression pattern of the above seven proposed circRNAs via qRT-PCR in PDAC tissues and adjacent normal tissues [67]. More recently, circRNAs expression in PDAC was explored by comparing PDAC tissues versus normal tissues by using microarray again. As a result, 256 differentially expressed circRNAs and 20 differentially expressed miRNAs were proposed to be associated with PDAC development [68].

Seimiya and coworkers [69] applied circular RNA-specific RNA sequencing and determined more than 40,000 previously unknown circRNAs that were altered in PDAC. Their research resulted in a novel circRNA, named circPDAC RNA, with no peptide production but the aberrant expression in PDAC tissues as well as patient serum. Another recent study involving a 208-case cohort of patients with PDAC identified a novel circRNA, named circBFAR or hsa_circ_0009065. The expression of circBFAR correlated positively with the tumor-node-metastasis stage and was related to the poor prognosis of patients with PDAC. Likewise, circBFAR knockdown dramatically inhibited the proliferation and motility of PDAC cells in vitro and their tumor-promoting and metastatic properties in the in vivo models [70]. A recent systematic review designating the roles of circRNAs in pancreatic and biliary tract cancers gathered detailed information and provided an understanding of the role of circRNAs in pancreatic cancer [71].

In recent studies, single-cell transcriptomics has paved the way to elucidate molecular biomarkers for early diagnosis of PDAC. Peng et. al. [72] found that a subset of ductal cells with unique proliferative features were associated with an inactivation state in tumor-infiltrating T cells, providing novel markers for the prediction of an antitumor immune response. EGLN3, MMP9, and PLAU have been reported as participating in PDAC carcinogenesis regarding dysregulated gene expression in malignant ductal cells [72]. In another single-cell RNA-sequencing study, sampling was from the mouse pancreas during the progression from preinvasive stages to tumor formation. While metaplastic cells were found to express two transcription factors, ONECUT2 and FOXQ1, the altered expression profiles of MARCKSL1, MMP7, and IGFBP7 were also observed, which could be accomplished as candidate markers for early detection of PDAC [72].

Consequently, findings provided by transcriptomic analysis of PDAC have been a valuable resource not only for deciphering the intra-tumoral heterogeneity and disease mechanism but also suggesting potential biomarkers for diagnosis, targeted therapy, or immunotherapy.

## 5. Proteomic Signatures of Pancreatic Cancer

Proteomics is a powerful approach that encompasses an extensive range involving the systematic analysis of protein structure, function, expression, protein–protein interactions, and posttranslational modifications [73]. Over many years, proteomics has been a key player for researchers to pinpoint biomarkers, which can be used as a tool for a faster disease diagnosis, prognosis, and enhanced treatment [74,75]. In terms of making contributions to clinical disease prediction, protein-based biomarkers are promising. The analysis and verification of unique protein biomarkers have been achieved by using highly sensitive and reliable mass spectrometry-based proteomics. Moreover, this technique is crucial in terms of querying protein modifications [20]. Numerous clinical specimens of pancreatic cancer such as pancreatic juice, pancreatic tumor tissue, pancreatic cyst fluid, urine, and plasma/serum have become targets for the proteomics field to dig into mechanisms of disease, improve novel biomarkers, and enhance drug development [76,77,78]. Identifying proteins or peptides detected in body fluids in cases of cancer might be useful for the early diagnosis of PDAC [78].

Sample type is a critical concern for the study of biomarkers. Since blood serum or plasma is convenient for periodic collections and includes a reproducible quantification, it is presumably the most preferred option. Although blood samples are easily accessible and noninvasive, the fundamental disadvantage of blood collection for the discovery of novel biomarkers is that not every protein carrying diagnostic potential is secreted into the bloodstream [79]. Investigation of the human pancreatic proteome has been done in patients with premalignant neoplasia, PDAC, and benign pancreatic disease. Although one of the most potent samples from the pancreas is the pancreatic juice, involving a high amount of proteins that might display the disease status, its collection is onerous since this procedure requires an endoscopy and cannulation of the pancreatic duct [80,81,82,83,84,85,86]. Collecting and conserving the intact tumor tissue and adjacent normal tissue is challenging due to the presence of digestive enzymes secreted by the pancreas. Nonetheless, pancreatic tissue is considered an excellent specimen for investigation of the pathological mechanisms underlying PDAC as well as for determining drug targets in virtue of its proximity to the lesion and its greater ingredient of tumor-related proteins [87]. Pancreatic cysts, which possess peculiarly stagnated fluids, are extensively seen as the most hopeful origin for the discovery of potential biomarkers since these tend to turn into pancreatic cancer [88]. In terms of urine, this is an effortlessly approachable biological specimen for biomarker detection, and its proteins are generated from both glomerular filtration and kidney [89]. Due to their accessibility and noninvasiveness, various urinary protein biomarkers have been examined to improve clinical assays for the diagnosis of several cancer types. As yet, merely a restricted amount of proteomics studies have been carried out to investigate the urinary proteome [90].

A retrospective study using a comprehensive proteomic analysis of pancreatic juice and pancreatic cell line samples from PDAC patients demonstrated that regenerating Family Member 1 Beta (REG1B) and syncollin (SYCN) could represent potential PDAC biomarkers [84,91]. Sogawa et al. [92] carried out a comparative proteomics analysis using a tandem mass tag (TMT) labeling and demonstrated that C4b-binding protein α-chain (C4BPA) is a novel serum biomarker for the early diagnosis of PDAC as well as for discrimination between PDAC and other gastroenterological cancers. Based on the results of a combinatorial proteomics strategy, Yoneyama et al. [93] indicated that insulin-like growth factor-binding proteins, IGFBP2 and IGFBP3, are compensatory biomarkers that can allow more accuracy through the combination with CA19-9 for the early detection of PDAC. In an MS-based proteomic study, Guo et al. [94] have demonstrated that dysbindin as a potential biomarker improved the accuracy of diagnosis in distinguishing PDAC from other pancreatic diseases. In a recent study, Cohen et al. [95] observed that the combination of testing circulating tumor DNA (ctDNA) with protein biomarkers (CA19-9, CEA, hepatocyte growth factor (HGF), and osteopontin) shows better performance than the CA19-9 test alone to distinguish PDAC from healthy controls. The improved accuracy of the biomarker panel—which is composed of a gold standard biomarker CA19-9, tissue factor pathway inhibitor (TFPI), and an isoform of tenascin C (TNC- FNIII-B)—in the differentiation of early-stage PDAC from different diseases was also demonstrated in a clinical cohort study [96]. In addition, Capello et al. [97] reported that the combination of TIMP1, LRG1, and CA19-9 performed better diagnostic accuracy than CA19-9 alone in differentiating early-stage PDAC from benign PDAC. Kim et al. [98] identified another biomarker panel that has high plasma THBS-2 and CA19-9 concentrations, which showed a remarkable differentiation ability between PDAC and healthy patients with 87% sensitivity and 98% specificity. The clinical significance of serum survivin was also reported in PDAC patients [99].

The pancreatic ductal fluid has been proposed as a good biological fluid for identifying prognostic biomarkers [100]. Focusing on the content of the ductal fluid, high concentrations of mucins and S100A8 or S100A9 were associated with the low survival rate in PDAC [100]. Ger et al. [101] recently investigated the proteome of 37 samples from pancreatic cancer and healthy subjects and identified that FLT3 and PCBP3 are promising prognostic biomarkers of pancreatic cancer.

Targeted proteomics is a rapidly evolving technological tool that conceptually represents an important advancement in alleviating the bottleneck in the preclinical biomarker assessment processes. In a targeted proteomics pilot study [102], five pancreatic cancer biomarker candidates—including 14-3-3 protein sigma, gelsolin, lumican, transglutaminase 2, and tissue inhibitor of metalloproteinase 1—were investigated in 60 plasma samples using a simple and robust selected reaction monitoring (SRM) multiplexed assay. Their results showed that gelsolin, lumican, and tissue inhibitor of metalloproteinase 1 have better area under curve (AUC) values than CA19-9 to discriminate pancreatic cancer from healthy controls and chronic pancreatitis controls. Yoneyama and colleagues [103] developed a quantification method specific for α-fibrinogen hydroxylated at proline residues 530 and 565 by SRM/multiple reaction monitoring (SRM/MRM). To validate these modifications as pancreatic cancer biomarkers, they quantified these posttranscriptional modifications in plasma samples from 70 pancreatic cancer patients and 27 healthy controls. They demonstrated that the plasma concentration of proline-hydroxylated α fibrinogen is significantly greater in pancreatic cancer patients.

In light of the rapidly developing accuracy and efficiency of proteomic approaches, our knowledge of the underlying molecular mechanism of pancreatic cancer has greatly increased [104,105]. However, there are still various limitations and analytic challenges that have resulted from the dynamic nature of the proteome of tissues and cells and the variation in the forms and functions of proteins due to several modifications [106]. Although several standardizations and improvements are required, proteomics is certainly a promising approach for the early diagnosis, prognosis, and discovery of targets for the treatment of pancreatic cancer.

## 6. Metabolomic Signature of Pancreatic Cancer

Metabolomics or metabolite profiling is a novel promising approach for the identification of robust biomarkers for diagnosis, prognosis, and assessment of treatment in pancreatic cancer [107,108,109,110,111]. Although there is currently no clinically validated metabolic biomarker that can help to provide early diagnosis of pancreatic cancer, the number of studies focusing on metabolic profiling and phenotyping of pancreatic cancer is increasing drastically [111,112,113,114]. As compared to other omics technologies, metabolic phenotyping is a sensitive indicator due to rapid and more precise results for new biomarker discovery [115]. The largest case-control study to discover a blood-derived metabolic biomarker signature that enables one to distinguish PDAC from chronic pancreatitis (ChP) was conducted by Mayerle et al. [114]. They investigated metabolomic profiles of plasma and serum samples from 914 subjects (patients with PDAC, ChP, liver cirrhosis, healthy, and non-pancreatic disease control), and a tumor biomarker signature (nine metabolites and additionally CA 19-9) was identified for differential diagnosis between PDAC and ChP with an AUC of 0.96. In a retrospective study investigating tissue metabolomics from 25 pancreatic cancer patients who had to undergo tumor resection surgery and gemcitabine-based adjuvant therapy, high lactic acid levels were observed in patients with poor clinical outcomes after gemcitabine therapy. Moreover, the combined evaluation of hENT1 with lactic acid showed superior performance in differentiating patients according to their overall survival [116]. In another study, Battini et al. [117] investigated tissue samples from 106 patients after PDAC resection to find metabolic biomarkers associated with long-term survival using metabolomic analysis methods. While the network analysis results revealed that higher levels of glucose, ascorbate, and taurine associated with long term survivors, decreased levels of choline, ethanolamine, glycerophosphocholine, phenylalanine, tyrosine, aspartate, threonine, succinate, glycerol, lactate, glycine, glutamate, glutamine, and creatine were estimated in long-term survivors. Due to the association of higher ethanolamine levels with worse survival, the metabolite with the highest accuracy in distinguishing between long-term and short-term survivors was ethanolamine.

An animal study was conducted to obtain metabolite profiling of pancreatic intraepithelial neoplasia (PanIN) and PDAC tissue samples from rats. They observed that the levels of kynurenate and methionine decreased in PDAC but increased in PanIN, demonstrating the potential of these metabolites to be biomarkers to differentiate PDAC from PanIN [116,118]. Laconti et al. identified that circulatory metabolite signatures can be used to differentiate animals with early-stage lesions with a diagnostic accuracy of 81.5% and 73.2% respectively [110].

Since the metabolic changes are quite important to detect and treat cancer regardless of the disease stage [119], genome-scale metabolic models (GEMs) might be a very helpful source to create and/or test the hypothesis for the elucidation of physiological mechanisms or novel biomarkers [120,121] so that GEMs can be used as a tool in both “top-down” and “bottom-up” methods in the context of biomarker discovery. GEMs have been employed for studying cancer metabolism utilizing either generic/personalized or tumor/cell-specific methods, which may translate into clinically relevant applications. They can also be used to identify drug targets leading to inhibition of cancer-related phenotypes or drug resistance in cancer therapy. Furthermore, the fortification of GEMs can be obtained via the integration of omics data like genomic, transcriptomic, and proteomic data, as well as the incorporation of regulatory molecules to the metabolism [122]. GEMs also provide valuable insight into the interaction between cancer cells and supporting cells in their niches as paving the way for whole-cell modeling [123,124].

In addition to all these, there are still some challenges in metabolomic studies. Whether significant changes in the metabolite level are due to the occurrence of the targeted disease, the use of non-confirmed metabolites with small sample size and the variability of patients’ parameters would affect the accuracy and reliability of the results [125]. Therefore, further standardization and improvement of currently available metabolomics techniques is a prospective requirement for the designation of highly accurate biomarkers that will provide significant clinical benefits and may help to obtain new target signatures for accurate diagnosis, imaging, and possible therapeutic options [126,127].

## 7. Glycomic Signatures of Pancreatic Cancer

Cancer studies are performed mostly based on alterations in genome, transcriptome, proteome, and metabolome levels, with a relatively small number of studies in alterations in glycan compositions and/or structures and glycoproteins [128]. However, the glycan studies have been increasing day by day to identify potential glycan alterations and glycoprotein biomarkers for cancer owing to the developments in glycans profiling [129]. In cancer cells, alterations in carbohydrate structures of secreted proteins are functionally significant and may offer promising targets to develop potential diagnostic and therapeutic strategies [130,131,132].

Since pancreatic cancer does not indicate any noticeable symptom during the early stages, it is a very difficult cancer type to diagnose [131]. It is an important challenge to detect new diagnostic biomarkers for pancreatic cancer. The glycoproteome occurring after co-translational or posttranslational modifications (PTM) and its role in the mechanism of pathogenesis have not been explained completely in pancreatic cancer. Besides, the available information about glycoproteome in normal pancreas and pancreatic cancer is very limited [133,134].

Glycosylation—the covalent attachment of a glycan to protein, lipid, carbohydrate, or other organic molecules—is the most common and complex PTM of proteins and significantly affects the function of proteins. Glycosylation of proteins plays an important role in various biological functions, including immune response and cellular regulation. Abnormal glycosylation is accepted as a molecular characteristic of transformation into malignant tumors for many epithelial cancers, including PDAC. Therefore, targeting aberrant glycosylation associated with cancer would be a useful approach to improve accurate diagnosis and possibly therapeutic strategies [129,133].

Several studies were published about glycan alterations and glycoproteome in pancreatic cancer. Pan et al. investigated protein N-glycosylation in pancreatic tumor tissue compared to the normal pancreas and chronic pancreatitis tissue through a quantitative glycoproteomics approach using HPLC and MS. This study presented a set of glycoproteins having aberrant N-glycosylation levels in pancreatic cancer, including mucin-5AC (MUC5AC), carcinoembryonic antigen-related cell adhesion molecule 5 (CEACAM5), insulin-like growth factor binding protein (IGFBP3), and galectin-3-binding protein (LGALS3BP) [133]. MUC5AC and CEACAM5 have been shown to play a role in tumor progression and metastasis in pancreatic cancer [133,135,136]. On the other hand, LGALS3BP was significantly hyperglycosylated in tumor tissue. Additionally, increased N-glycosylation on many cancer-associated aberrant glycoproteins was reported on pancreatic cancer-associated pathways such as TGF-β, TNF, NF-kappa-B, and TFEB-related lysosomal changes [133].

Yue et al. studied sera from pancreatic cancer patients to determine certain glycan alterations and their possible usage in the diagnosis of pancreatic cancer. To that end, they characterized glycan and protein levels of specific mucins and carcinoembryonic antigen-related proteins of these patients through the antibody-lectin sandwich array method previously developed. They found that MUC16 protein was frequently increased (65% of the patients) in the cancer patients, whereas MUC1 (30%) and MUC5AC (35%) proteins were less frequently elevated. In addition to this, MUC1 and MUC5AC proteins indicated highly extensive and diverse glycan alterations, while MUC16 protein did not. The most frequent glycan elevations that affected these proteins involved the Thomsen–Friedenreich antigen, fucose, and Lewis antigens. Additionally, they reported an unanticipated enhancement in the exposure of alpha-linked mannose on MUC1 and MUC5AC. Moreover, the CA19-9 on MUC1 had the most important increase (87%) in cancer patients with 4% of the control subjects [130].

In another study, N-glycosylation at Asn88 in serum human pancreatic ribonuclease 1 (RNase1) was substantially elevated in pancreas cancer patients compared with normal human subjects [131]. Similarly, increased fucosylation levels of serum α-1-acid glycoprotein (AGP) glycoforms were reported in pancreatic cancer compared to healthy controls and pancreatitis patients via numerous analytical methods consisting of MS, capillary zone electrophoresis (CZE), and enzyme-linked lectin assays (ELLA) [134].

As an alternative therapy option having fewer adverse effects than others, regional intra-arterial chemotherapy (RIAC) is preferred for advanced pancreatic cancer. Qian and colleagues [137] took advantage of the presence of Glypican-1 (GPC1) in extracellular vesicles (EVs) to determine if the change in GPC1+ cells in EVs could be a predictor of the consequences of RIAC for advanced pancreatic cancer patients. They concluded that patients with advanced pancreatic cancer who displayed a decrease in GPC1+ EVs experienced enhanced overall survival rates with the aid of RIAC therapy.

Another cell-surface glycoprotein, CD44 is a known prognostic biomarker and therapeutic target in pancreatic cancer [138]. The overexpression of CD44 was shown to be associated with aggressive malignant attitudes, cell migration, and distance metastasis, therefore with poor overall survival in patients with pancreatic cancer [138]. On the other hand, the reduction in CA19-9 levels envisaged a good prognosis after neoadjuvant therapy with a low incidence of recurrence after surgery [139].

All of these studies provide an insight into the potential biomarker candidates for effective diagnosis, prognosis, and treatment in pancreas cancer using measurements in glycan alterations on precise glycoproteins.

## 8. Metagenomic Biomarkers of Pancreatic Cancer

In recent studies, the interaction between microbiomes and the initiation and progression of pancreatic cancer has become recognized, raising the possibility of identifying novel diagnostic and prognostic factors for PDAC [140]. The existence of intratumoral microbiota is considered to have a potential etiologic impact on pancreatic carcinogenesis, including inflammation, immunosuppression, and stimulation of cellular carcinogenic pathways [141,142,143].

It is becoming clear that there is a correlation between oral microbiota and PDAC, and the abnormalities of oral microbiota have been proposed to appear before the development of cancer [144]. Available literature data provide knowledge on the oral bacteria that might play a pathogenic role in the progression of PDAC, and these are *Porphyromonas gingivalis*, *Fusobacterium*, *Neisseria elongata*, and *Streptococcus mitis* [145]. In this context, a large metagenomic study comparing PDAC patients and healthy controls revealed that *P. gingivalis* was associated with an approximately 60% greater risk of PDAC [146]. Mitsuhashi et al. [147] indicated that the existence of approximately 10% *Fusobacterium* in pancreatic cancer tissue is independently associated with poor prognosis of PDAC but not with its clinical and molecular features. It is also thought that *Fusobacterium* species may be a candidate prognostic biomarker for pancreatic cancer and should be considered for further oral microbiota studies. On the other hand, some studies have revealed that *Fusobacteria* are associated with reduced risk of PDAC, revealing that the role of *Fusobacteria* on PDAC could be controversial [144,146,148].

Fecal microbial transplantation (FMT) possesses an enormous amount of microbiota compared to usually preferred probiotic supplements and might provide a significant movement in reducing the immunosuppression and in increasing the response rate to treatment in cancer patients having a probable low survival [149]. In a recent cohort study, Riquelme and colleagues [150] made a metagenomic analysis from 68 tumor samples of tumor microbiome composition of PDAC patients with short-term survival (STS) and long-term survival (LTS) phenotypes using 16S rRNA gene sequencing. They reported that the tumor microbiome diversity of long-term survivors was higher than that of short-term survivors, potentially representing a strong interrelation between the gut microbiome and patients’ survival rate. Besides, animal studies by human-into-mice FMT experiments from STS, LTS, and healthy donors conspicuously confirmed that the transference of the long-term survivors’ gut microbiome can modulate the intratumoral microbiome. According to a study encompassing a comparative analysis of fecal microbiota from PDAC patients and control donors in murine models, a certain type of bacteria—namely, Proteobacteria, Actinobacteria, Fusobacteria, and Verrucomicrobia—are found in higher amounts in the gut of PDAC patients. Specifically, the gut microbiota of PDAC patients contains greater amounts of Proteobacteria (45%), Bacteroidetes (31%), and Firmicutes (22%). This study remarkably highlights that the intratumoral microbiome associated with pancreatic cancer has relatively distinct proportions in comparison to the microbiome of normal pancreatic tissue [143].

In an animal study, Mendez et al. [151] demonstrated a substantial correlation between microbial dysbiosis and the release of tumor-inducing metabolites in the early-stage, while showing significantly elevated serum polyamine concentrations in PDAC patients; this may be postulated as a predictive biomarker for early detection of pancreatic cancer. It is among the current assumptions that bacteria in the pancreatic microbiome may contribute to the resistance of gemcitabine, which is widely used in the treatment of PDAC. Based on this assumption, 76% of the tested pancreatic tissue was found to be positive for bacteria, particularly Gammaproteobacteria [152].

Several studies also suggest that the composition of oral [146,148,153], fecal [154], and pancreatic microbiome [143,155] may be used for early diagnosis of PDAC. With the accumulation and advanced evaluations of data on the pancreas, gut, and oral microbiota, it might be possible to develop microbiome screening methods that can be considered as a promising tool in the prediction of PDAC risk and treatment of disease progression.

## 9. Biomarkers Leading to Improved Personalized Medicine

On the way to personalized medicine, there are promising and on-going efforts for the integration of multi-omic data. As an aim of precision medicine, the first attempt is to stratify patients according to their disease subtypes, biomarkers, clinical features, or demography. Later, in addition to the stratification process, more features such as environment, medication history, behaviors, and habits are utilized to create smaller groups. In theory, this stratification technique should avoid failures in clinical trials since the suitable diagnosis and targeted treatments are applied to small patient populations or directly to individuals. Instead of “one-size-fits-all” treatment approaches, the best therapy options or medications for each individual or a small group can be achieved through disease stratification and then personalization by the integration of multi-omics networks. In addition, personalized medicine treatment necessitates the co-development of diagnostic tools (preferably within noninvasive methods) to characterize the ideal therapy for patients. There is an urgent need for multi-omic data integration not only for pancreatic cancer but also for many other diseases from the personalized medicine perspective in the future (Figure 1).

According to the present clinical data, using only chemotherapeutic approaches in the treatment of pancreatic cancer will likely be insufficient in terms of the increase in survival time and response rate in the near future. Therefore, there is an urgent need for precision medicine, which aims at tailoring the best treatment option for individual patients based on their genomic information, together with molecular, environmental, and lifestyle factors, to identify the suitable biomarkers and targeted therapies for cancer patients. Personalized medicine stratifies the patients by considering the individual differences among cancer patients, unlike conventional therapy. As in other types of cancer, studies on precision medicine in pancreatic cancer have increased in recent years [156,157].

There are several precision medicine programs and clinical studies run by various initiatives from different countries to offer the best personalized treatment options for pancreatic cancer patients according to their molecular tumor profiling [156]. These programs have demonstrated that a small patient cohort had better progression-free survival after switching their therapies from standard-of-care treatment to molecular-targeted therapy [158]. Further, molecular profiling of tumors from patients with all stages of pancreatic cancer was performed using NGS to develop response rates and therapeutic biomarkers [159]. Besides, different clinical studies were performed to discover biomarkers for prognosis or treatment response [160], focusing on alterations in genome and epigenome in tumor tissue [161]. The Comprehensive Molecular Characterization of Advanced Pancreatic Ductal Adenocarcinoma for Better Treatment Selection (COMPASS) trial was the prospective translational study that investigated the feasibility of comprehensive real-time genomic analysis of advanced PDAC, integrating genomic and transcriptomic subtypes and chemotherapy response [162].

The alterations in the genome, epigenome, proteome, and metabolome cause the changes in the phenotype in pancreatic cancer, and thus studies carried out on these alterations could help with the stratification of pancreatic cancer. The identification of new biomarkers for subtyping, diagnosing cancer, and predicting therapy response is an ongoing process in preclinical studies. However, the difficulties in the translation of promising preclinical findings into clinical practice make the application of precision medicine approaches in clinics a great challenge. These difficulties arise from the evaluation of basic science findings in the clinical settings and the selection of the best effective scientific data for clinical trials [156]. Moreover, it is very important and vital to building collaborations among basic scientists, clinicians, and bioinformaticians to overcome these challenges.

For patients with pancreatic cancer, CA19-9 is the only routinely used serum biomarker in prognosis and early diagnosis of recurrence after therapy [156]. Although the increase in CA19-9 level indicates advanced pancreatic cancer and poor prognosis [139], this elevation can be only observed in 65% of the patients with resectable pancreatic cancer, in addition to patients with other diseases such as pancreatitis or cirrhosis [163]. Besides, 10% of patients with pancreatic cancer cannot synthesize CA19-9 even if they are in the advanced stage, since they are negative for Lewis antigen a or b. Moreover, it is not a screening biomarker for pancreatic cancer to be used alone [156].

Numerous gene alterations that play important roles in tumorigenesis can provide the development of novel treatments that target specific genes for pancreatic cancer patients. Personalized medicine can certainly improve the management of patients and outcomes of novel treatments with the administration of the right therapy using the right dose at the right time to the right patient when applied to pancreatic cancer patients. The generation of well-designed clinical trials allowing the construction of molecular profiling of tumors of patients will further guide the development of novel and effective strategies for the overall survival of patients in this highly lethal cancer [157,160].

## 10. Conclusions

There are big initiatives, various research programs, and databases in which researchers are able to collect different omics datasets of pancreatic cancer. However, many biomarker studies have been challenged by low case numbers, non-specificity of molecular markers and their low reproducibility, and the absence of preclinical or clinical as well as feasibility studies.

The well-known example of pancreatic cancer biomarkers is CA19-9, but as a single biomarker it cannot offer a potential to be used in the clinic. Recent studies on non-coding RNAs such as miRNAs, circRNAs, and lncRNAs hold great promise not only as biomarkers but also for understanding the regulatory network components in pancreatic cancer. Targeted or shotgun proteomic approaches also provide an opportunity for more sensitive or novel biomarker identification. Metagenomics is another emerging technique that measures altered microorganism abundance and may act as a potential biomarker. On the other hand, although the pancreas is at the center of many metabolic pathways, the metabolic rewiring of pancreatic cancer is an underestimated topic since the number of metabolomics studies are not as numerous as some of the other omics investigations.

Although many novel markers have been discovered through omics studies of PDAC in the past decade, none of those novel biomarkers have yet been brought into routine clinical practice. However, there is a hope that various combinations of these biomarkers as a biomarker panel may result in a clinical output, and this fact makes the integration of multi-omics data more challenging on the way to translating omics markers into the clinic.

Another point that has a crucial role in translation to the clinic is sampling, where body fluids are favorable for the detection of the biomarkers. Later, these biomarkers also assist oncologists in deciding optimal therapeutic management by defining the way for precision treatment.

In conclusion, there is great attention focusing on multi-omics biomarkers in terms of their diagnostic, predictive, and prognostic potentials to fight against pancreatic cancer as well as other cancer types. One of the major medical concerns raised by oncologists is the identification of robust, reasonable, and reliable diagnostic biomarkers since early detection of pancreatic cancer is crucial for personalized therapy options and improved survival outcomes. This strategy can be accomplished by a systems biology approach that aims to organize multi-omics data despite the challenges. Successfully accomplishing multi-omics data integration by systems biology approaches will fulfill future expectations and the need for robust, accurate, and feasible biomarker panels for pancreatic cancer.

**Table 1:** A summary of methodology and sampling used in biomarker studies for pancreatic cancer.

“Omic” Level Description		Sample Origin	Altered Molecule/Microorganism	Expression Pattern	Detection Method *	Reference Study
Genomics	Mutation	Pancreatic tissue	CDKN2A, CDKN2B, TP53, SMAD4, KRAS	-	WES/WGS	[28]
Transcriptomics	Coding RNAs	T cell	EGLN3, PLAU	Downregulated	scRNA-seq	[72]
		T cell	MMP9	Dysregulated	scRNA-seq	[72]
		Mouse pancreatic tissue	ONECUT2, FOXQ1, MARCKSL1, MMP7, IGFBP7	Upregulated	scRNA-seq	[164]
		Tumor tissue	hsa_circ_100782	Upregulated	Microarray/qRT-PCR	[71]
	circRNAs	Tumor tissue/plasma/cell lines	hsa_circ_0006988	Upregulated	qRT-PCR	[165]
		Tumor tissue/cell lines	hsa_circ_0099999 (circZMYM2)	Upregulated	circRNA overexpression	[166]
		Tumor tissue	hsa_circ_0006215	Upregulated	circRNA overexpression	[167]
		Tumor tissue, plasma exosome	circ-IARS	Upregulated	circRNA overexpression	[168]
		Tumor tissue	circ-PDE8A	Upregulated	circRNA overexpression	[169]
		Tumor tissue/cell	hsa_circ_0001649	Downregulated	Microarray/qRT-PCR	[170]
		Tumor tissue/cell	hsa_circ_0005397 (circ-RHOT1)	Upregulated	Microarray/qRT-PCR	[171]
		Tumor tissue/cell lines	hsa_circ_0030235	Upregulated	circRNA overexpression	[172]
		Tumor tissue/cell lines	hsa_circ_0007534	Upregulated	circRNA overexpression	[173]
		Tumor tissue/cell lines	ciRS-7 (Cdr1as)	Upregulated	qRT-PCR	[174]
		Tumor tissue	hsa_circ_0007334	Upregulated	Microarray/qRT-PCR	[175]
		Tumor tissue	circLDLRAD3	Upregulated	circRNA knockdown	[176]
		Tumor tissue/cell	circASH2L	Upregulated	Microarray/qRT-PCR	[177]
		Tumor tissue/cell lines	circADAM9	Upregulated	circRNA knockdown	[178]
		Tumor tissue/cell	hsa_circ_001653	Upregulated	circRNA knockdown	[179]
		Tumor tissue/cell	circHIPK3	Upregulated	circRNA knockdown	[180]
		Tumor tissue/cell	circFOXK2	Upregulated	circRNA knockdown	[181]
		Tumor tissue	hsa_circ_0009065 (circBFAR)	Upregulated	circRNA overexpression	[70]
		Tumor tissue	hsa_circ_0086375 (circNFIB1)	Downregulated	circRNA knockdown	[182]
		Tumor tissue/cell	hsa_circ_0013912	Upregulated	circRNA overexpression	[183]
		Tumor tissue/cell lines	hsa_circ_001587	Downregulated	circRNA knockdown	[184]
		Tumor tissue	hsa_circ_0001946, hsa_circ_0005397	Upregulated	Microarray/qRT-PCR	[67]
		Tumor tissue	hsa_circ_0005785, hsa_circ_0006913, hsa_circ_0000257, hsa_circ_0041150, hsa_circ_0008719	Downregulated	Microarray/qRT-PCR	[67]
		Plasma	miR-21	Upregulated	Microarray/qRT-PCR	[49]
		Pancreatic juice	miR-155	Upregulated	qRT-PCR	[49]
	miRNAs	Tumor tissue/cell lines	miR-196a	Upregulated	Microarray/qRT-PCR	[185]
		Tumor tissue	miR-210	Upregulated	qRT-PCR	[186]
		Tumor tissue/cell line/serum	miR-1290	Upregulated	Microarray/qRT-PCR	[50]
		Tumor tissue/cell lines	miR-200a/miR-200b	Upregulated	Microarray/qRT-PCR	[51]
		Tumor tissue/plasma/serum	miR-18a	Upregulated	qRT-PCR	[55]
		Tumor tissue	miR-192	Upregulated	Microarray/qRT-PCR	[187]
		Blood	miR-22-3p/miR-642b/miR-885-5p	Upregulated	qRT-PCR	[188]
		Tumor tissue	miR-23a/miR-31/miR-100/miR-143/miR-221	Upregulated	qRT-PCR	[43]
		Tumor tissue	miR-148a/miR-375/miR-217	Downregulated	qRT-PCR	[43]
		Plasma	miR-16 and miR-16 and miR-196a and CA 19-9 combination	Upregulated	qRT-PCR	[56]
		Peripheral Blood Mononuclear Cells	miR-27a-3p with CA 19-9	Upregulated	RNA-seq/qRT-PCR	[57]
		Tumor tissue/cell lines	miR-221/miR-222	Upregulated	qRT-PCR	[185]
		Tumor tissue/plasma	miR-744	Upregulated	Microarray/qRT-PCR	[62]
		Tumor tissue	miR-218	Downregulated	Microarray/qRT-PCR	[189]
		Tumor tissue	miR-494	Downregulated	Microarray/qRT-PCR	[46]
		Tumor tissue	HOTAIR	Upregulated	qRT-PCR	[35]
		Tumor tissue	PVT1	Upregulated	qRT-PCR	[190]
	Other ncRNAs	Tumor tissue	MALAT-1	Upregulated	qRT-PCR	[191]
		Tumor tissue	Gas5	Upregulated	qRT-PCR	[192]
		Tumor tissue	MEG3	Upregulated	qRT-PCR	[193]
		Tumor tissue	HULC	Upregulated	qRT-PCR	[194]
		Tumor tissue	BC008363	Upregulated	Microarray/qRT-PCR	[195]
		Tumor tissue	HSATII	Upregulated	RNA-seq	[196]
		Serum/plasma	U2snRNA	Upregulated	Microarray/qRT-PCR	[197]
		Pancreatic juice and cell line	REG1B/SYCN	Upregulated	ELISA	[84]
		Serum	C4BPA	Upregulated	TMT labeling	[92]
Proteomics	Proteins	Plasma	IGFBP2/IGFBP3	Upregulated	Antibody-based and LC-MS/MS-based	[93]
		Serum	DTNBP1	Upregulated	MS	[94]
		Plasma	ctDNA with CA19-9, CEA, HGF, and osteopontin	Upregulated	Luminex bead-based immunoassays	[95]
		Plasma	Combination of CA19-9, TFPI, and TNC- FNIII-B	Upregulated	ELISA	[96]
		Plasma	Combination of TIMP1, LRG1, and CA19-9	Upregulated	ELISA	[97]
		Plasma	THBS-2 and CA19-9	Upregulated	ELISA	[98]
		Serum	Survivin	Upregulated	ELISA	[99]
		Pancreatic ductal fluid	Mucins and S100A8 or S100A9	Upregulated	MS	[100]
		Tumor tissue	FLT3, PCBP3	Upregulated	HDMS	[101]
		Tumor tissue	Combination of hENT1 and lactic acid		GC/TOF-MS	[116]
		Tumor tissue	Glucose, ascorbate, ethanolamine, and taurine	Upregulated	HRMAS-NMR	[117]
		Tumor tissue	Choline, ethanolamine, glycerophosphocholine, phenylalanine, tyrosine, aspartate, threonine, succinate, glycerol, lactate, glycine, glutamate, glutamine, and creatine	Downregulated	HRMAS-NMR	[117]
Metabolomics	Metabolites	Rat tumor tissue	Kynurenate and methionine	Downregulated	NMR	[116]
		Tumor tissue	N-glycosylation of MUC5AC, CEACAM5, IGFBP3, and LGALS3BP	Upregulated	HPLC, MS	[133]
		Serum	α-linked mannose and glycan involved the Thomsen–Friedenreich antigen, fucose, and Lewis antigens affected MUC1 and MUC5AC	Upregulated	Microarray, WB	[130]
		Serum	Asn-88 N-glycosylation and differential RNase-1 expression	Upregulated	ELISA, WB	[131]
Glycomics	Glycan alterations	Serum	α1-3 fucosylation in α-1-acid glycoprotein	Upregulated	ELLA, HILIC-MS, CZE	[134]
		Serum	CA19-9	Downregulated	Immunoassay	[139]
		Tumor biopsy	CD44 antigen (CD44)	Upregulated	WB	[138]
		Plasma	Glypican-1 (GPC1)	Upregulated	Flow cytometry	[137]
	Glycoproteins	Serum	Mucin-5AC, MUC1, and MUC16	Upregulated	Antibody-lectin sandwich array	[130]
Metagenomics	Microbiota	Oral microbiota	*Porphyromonas gingivali*, *Fusobacterium*, *Neisseria elongata*, and *Streptococcus mitis*	High amount	plasma antibody analysis, 16S rRNA sequencing	[145]
		Murine fecal microbiota	Proteobacteria, Actinobacteria, Fusobacteria, and Verrucomicrobia	High amount	qPCR, FISH, 16S rRNA gene sequencing	[143]
		Murine gut microbiota	Proteobacteria, Bacteroidetes, and Firmicutes	High amount	qPCR, FISH, 16S rRNA gene sequencing	[143]

* CZE: capillary zone electrophoresis, ELISA: enzyme-linked immunosorbent assay, ELLA: Enzyme-linked lectin assay, FISH: fluorescence in situ hybridization, GC: gas chromatography, HILIC: Hydrophilic interaction chromatography, HRMAS: high-resolution magic angle spinning, LC: liquid chromatography, MS: mass spectrometry, NMR: nuclear magnetic resonance, qRT-PCR: quantitative reverse transcription polymerase chain reaction, TOF: time of flight, WB: Western blot, WES: Whole exome sequencing, WGS: whole genome sequencing.
